# Online and Ex Situ Damage Characterization Techniques for Fiber-Reinforced Composites under Ultrasonic Cyclic Three-Point Bending

**DOI:** 10.3390/polym16060803

**Published:** 2024-03-13

**Authors:** Aravind Premanand, Mario Prescher, Michael Rienks, Lutz Kirste, Frank Balle

**Affiliations:** 1 Department for Sustainable Systems Engineering (INATECH), Faculty of Engineering, University of Freiburg, 79110 Freiburg, Germany; michael.rienks@inatech.uni-freiburg.de (M.R.); frank.balle@inatech.uni-freiburg.de (F.B.); 2 Fraunhofer Institute for Applied Solid State Physics (IAF), 79108 Freiburg, Germany; mario.prescher@iaf.fraunhofer.de (M.P.); lutz.kirste@iaf.fraunhofer.de (L.K.); 3 Freiburg Materials Research Center (FMF), 79104 Freiburg, Germany; 4 Fraunhofer Institute for High Speed Dynamics, Ernst Mach Institute (EMI), 79104 Freiburg, Germany

**Keywords:** ultrasonic fatigue testing, carbon fiber reinforced polymers, online monitoring, fatigue damage, X-ray microscopy

## Abstract

With ultrasonic fatigue testing (UFT), it is possible to investigate the damage initiation and accumulation from the weakest link of the composite material in the very high cycle fatigue (VHCF) regime in a shorter time frame than conventional fatigue testing. However, the thermal influence on the mechanical fatigue of composites and the scatter in fatigue data for composites under ultrasonic cyclic three-point bending loading still need to be investigated. In this study, we conducted interrupted constant-amplitude fatigue experiments on a carbon-fiber satin-fabric reinforced in poly-ether-ketone-ketone (CF-PEKK) composite material. These experiments were carried out using a UFT system, which operates at a cyclic frequency of 20 kHz with a pulse–pause sequence. Various parameters, such as the CF-PEKK specimen’s surface temperature, acoustic activity, and the ultrasonic generator’s input resonance parameters, were measured during cyclic loading. During experiment interruption, stiffness measurement and volumetric damage characterization in the CF-PEKK specimens using 3D X-ray microscopy (XRM) were performed. The locations of damage initiation and accumulation and their influence on the changes in in situ parameters were characterized. Under fixed loading conditions, damage accumulation occurred at different locations, leading to scattering in fatigue life data. Further, the damage population decreased from the surface to the bulk of the composite material.

## 1. Introduction

Continuous fiber-reinforced polymer matrix composites (PMCs) with textile reinforcements are considered an essential class of high-performance materials as they exhibit improved damage tolerance behavior compared to unidirectional (UD) cross-ply laminates [1]. Other advantages of woven-fabric composites include high toughness, dimensional stability for various temperatures, and simple manufacturing [2]. Reliable designs of structures made out of carbon fabric-reinforced polymers (CFRPs) require an accurate evaluation of their fatigue life under service loading conditions [3]. Two notable applications for textile composites are aerospace [4] and wind energy engineering [5].

During their service life of up to 30 years, aerospace and wind energy industry structures are often loaded with more than $10^{8}$ cycles [6]. Many material configurations resulting from the fibers, fiber architectures, matrices, manufacturing methods, as well as stacking sequences make the generic fatigue characterization difficult [7]. The loading directions in the structures also have a strong influence on the fatigue of composites. When the stress state is not simply tensile, uni-axial, and aligned with the fibers, severe loads are placed on the matrix, reducing the composite’s fatigue resistance [8]. To fully utilize the mechanical properties of CFRPs for lightweight applications, the fatigue behavior and underlying failure mechanisms must be well understood.

### 1.1. Fatigue Damage in Woven Fabric-Reinforced Composites

The main fatigue damage mechanisms observed during fatigue loading of woven-fabric composites can be classified into micro-structural damage within the strand of fibers and macroscopic damage across the different layers. The micro-structural damage mechanisms include matrix micro-cracks, fiber-matrix debonding, fiber breakage, and crack coupling, synonymous with laminated composites built up of UD layers [2]. The macroscopic damage mechanisms include transverse cracks in the weft fiber bundles, shear failure in the wrap fiber bundles, cracks in matrix regions, delamination between weft and warp fiber bundles, delamination between adjacent fabric layers, tensile failure of warp bundles, and final fracture [2]. Due to the interlacing fiber architecture in woven-fabric composites, the damage accumulation is gradual until the start of the final phase of fatigue life. Further, the textile reinforcements in woven composites limit the growth of cracks and delamination compared to UD reinforcements [9]. Compared to UD laminates, the main damage mechanisms due to fatigue of woven laminates are transverse cracks, delaminations, and fiber failure [10,11,12,13,14]. Lamon et al. (2023) [15] observed that one of the main damage mechanisms in woven fiber architecture before final failure is the initiation and propagation of off-axis cracks (transverse cracks) in the fiber bundles. During the final phase of damage growth, all the damage modes can interact and grow rapidly. The final failure is usually marked by the fracture of warp bundles, leading to the ultimate failure of the laminate.

Most of the work on fatigue damage analysis of woven composites has been performed in low- and high-cycle fatigue regimes of composites, and the limited knowledge of very high cycle fatigue (VHCF) is compensated by conservative designs [16]. The limited works in the VHCF regime can be explained by the very long test periods to realize experiments beyond $10^{7}$ cycles. Accelerated fatigue testing methods, where the cyclic frequency is increased, offer a potential solution to reduce the experimental time to reach the VHCF regime. Various authors have studied different CFRP composites since the first cyclic three-point bending test setup was developed for conducting VHCF experiments in an ultrasonic fatigue test system for composites [17]. A carbon fiber-reinforced in polyphenylene sulfide (CF-PPS) composite system stacked with 2/2 twill fabric in an orthotropic layup exhibited a five-stage damage accumulation starting from fiber matrix debonding coalescing to transverse cracks and micro-delaminations between 0° and 90° fiber rovings. This damage mechanism was followed by the development of meta- and macro-delaminations, which led to the final failure of the short beam specimens [18,19]. In contrast, during another study on a CF–epoxy composite system stacked with 4-Harness satin fabric in a quasi-isotropic layup, sudden failure by fracture at the bottom of the specimens was observed [20]. A CF–epoxy composite system with plain weave fabric stacked with alternating 0° and 90° layers was reported to undergo pitting corrosion with damages in the epoxy matrix [21].

### 1.2. Damage Monitoring Techniques

Different non-destructive techniques have been applied to detect and evaluate fatigue damage in composites. This includes ultrasonic C-scans for evaluation of delamination onset and growth [22,23,24], acoustic emission technique to capture material degradation and fatigue damage evolution [25,26,27,28], and thermography [29,30,31,32]. The use of X-ray computed tomography (X-CT) as a non-destructive inspection technique for assessing damage in composites has increased in the last decade [33]. This is mainly due to the possibility of volumetric damage characterization with a sufficient resolution (up to 1 μm) [34]. Yu et al. (2016) [35] performed X-ray tomography at different stages of fatigue life for 3D woven glass fiber-reinforced composite specimens by performing interrupted axial fatigue experiments. Jespersen et al. (2016) [36] studied the progression of fatigue damage in a uni-directional, non-crimp glass fabric-reinforced composite under tension–tension loading. Here, the damage progression as a function of stiffness degradation was examined by CT scans on four different samples, each at different phases: one during the initial drop, two during stable stiffness degradation, and one close to final failure. Later, Djabali et al. (2019) [37] investigated the fatigue damage mechanisms in thick CF–epoxy laminate subjected to bending loads by combining in situ monitoring techniques such as acoustic emission (AE) and digital imaging correlation (DIC) and X-CT. This combination enabled the identification and quantification of fatigue damage.

### 1.3. Motivation for Current Work

The knowledge and experience on fatigue of composites in the VHCF regime conducted using a UFT system under three-point bending by the authors [38] as well as others [18,20,21] are limited to the surface of the composite specimens. Under a pure bending load case, maximum cyclic compressive stresses occur at the top layer of the composite specimen, and tensile stresses occur at the bottom layer. However, since the resonance condition for a three-point bending load case leads to short specimens, typically in the order of 30–35 mm for carbon fiber-reinforced polymer composites, cyclic shear stresses occur in the regions between the loading nose and support units. Light optical or scanning electron microscopy (used in [18,20,21,38]) can provide excellent results on the state of damage anywhere in the specimen but is limited to only one plane. Specimens have to be physically sliced to acquire information across multiple planes. The goal of this investigation was to determine the volumetric damage state of the composite specimens due to ultrasonic cyclic loading and relate it with the existing damage monitoring techniques that are already used for UFT.

## 2. Material and Specimen Geometry

Recently, the applicability of the CF-PEKK composite material was demonstrated by the Thermoplastic Affordable Primary Aircraft Structure (TAPAS) Consortium for small and large aircraft structures [39,40,41,42]. PEKK is a semi-crystalline thermoplastic polymer with a glass transition temperature ($T_{g}$) of ≈160 °C [43] and a processing temperature slightly lower than that of PEEK [44]. Thus, the carbon fiber five-harness satin fabric-reinforced poly-ether-ketone-ketone (CF-PEKK) composite material was chosen for the current study. The composite laminates were manufactured by Toray Advanced Composites (Nijverdal, The Netherlands). The physical properties of the CF-PEKK composite laminate provided in Table 1 were already published in [38,45].

The dimensions of the composite specimens have to be evaluated using modal analysis to conduct cyclic loading experiments in ultrasonic fatigue testing systems. The evaluated dimension enables the composite specimens to be stimulated with resonance vibrations in the desired eigenmode at a frequency of 20 kHz [17]. The dimensions of the cyclic three-point bending specimen for testing the CF-PEKK material at a cyclic frequency of 20 kHz were previously determined during an existing study [46]. The specimen dimensions were 34 × 15 × 4.1 mm^3^ [46].

For determining the flexural properties of fiber-reinforced polymers under three-point bending, ASTM D7264/D7264M-15 [47] recommends a minimum span-to-thickness ratio of 16:1. However, the obtained specimen geometry results in a span-to-thickness ratio of 4.5:1. This produces flexural as well as shear stresses during cyclic loading. However, in a previous investigation by the authors on the CF-PEKK specimens [46], monotonic or quasi-static experiments failed in the middle of the specimen due to normal stresses. Thus, normal stresses were evaluated for CF-PEKK specimens for the current span-to-thickness ratio [48].

The lateral movement during resonance oscillations can be avoided by accurately positioning the specimens with the support pins at the nodal points where displacements are zero during transverse bending eigenmode [17]. For the CF-PEKK specimen, the span length or the distance between the support pins was 18.6 mm [46]. Another study experimentally validated the desired oscillation mode during ultrasonic cyclic loading for the CF-PEKK specimens using 3D Scanning Laser Doppler Vibrometry (3D-SLDV) from Polytec GmbH (Waldbronn, Germany) [48].

## 3. Methodology

### 3.1. Description of the UFT System

**Figure 1:**
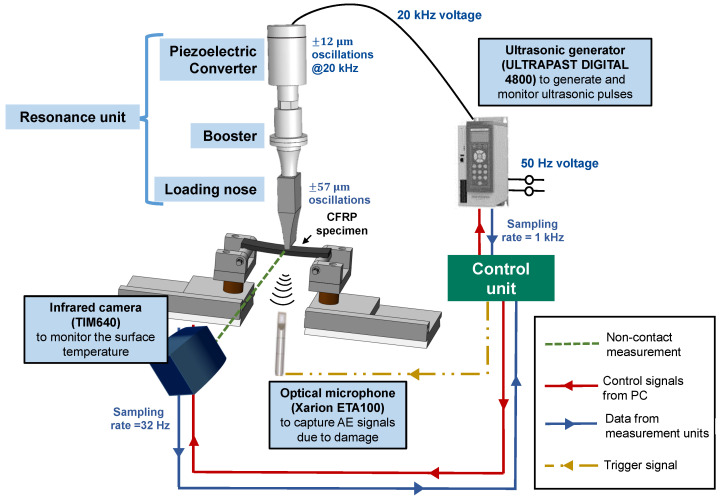
A schematic of the experimental setup showing ultrasonic fatigue testing and integrated and online monitoring systems for performing cyclic three-point bending loads on CFRP specimens.

**Figure 2:**
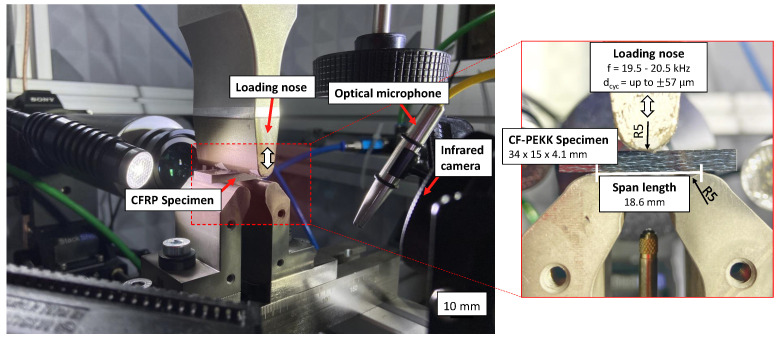
The ultrasonic fatigue testing system for three-point bending loading of CF-PEKK specimens.

An in-house UFT system was developed using the ultrasonic load train manufactured by Herrmann Ultraschalltechnik GmbH (Karlsbad, Germany) at INATECH, University of Freiburg. A schematic of the loading system and the online monitoring units, along with their model names, are provided in Figure 1. A mechanical oscillation amplitude up to 57 μm can be achieved with this loading setup and the resonance unit. This is shown in Figure 2. Consequently, the fatigue experiments are displacement-controlled. A control unit is realized using LabVIEW 2018 software in a computer with the Windows operating system. Further information about the resonance train can be found in [48]. The developed LabVIEW interface allows for the user to

- control and record the ultrasonic generator’s input resonance parameters at a sampling rate of 1 kHz [38,49],
- send the trigger signals to the optical laser microphone to record the acoustic activity, and
- capture the maximum surface temperature on the CF-PEKK specimen at a sampling rate of 32 Hz [38,45,49].

Two data acquisition systems (DAQ) from National Instruments were integrated with the control unit to realize high-speed data transfer between the ultrasonic generator, the online monitoring units, and the computer with the Windows operating system. Compressed air cooling was realized during the entire duration of the fatigue experiments, with four nozzles blowing air on all four sides of the specimen.

### 3.2. Online Monitoring Techniques

During the fatigue experiments, the resonance data for every ultrasonic pulse can be fetched at the end of each ultrasonic pulse from the ultrasonic generator. These data include input displacement, generator power, and resonance frequency. The evolution of the maximum surface temperature of the specimen due to ultrasonic oscillations was monitored using an infrared (IR) camera of type TIM640 from Micro-Epsilon Messtechnik GmbH (Ortenburg, Germany) at a frame rate of 32 Hz. Up to 4 areas can be defined on the surface of the specimens, and the maximum temperature as a function of time is recorded at the sampling frequency of the IR camera for the defined areas. The setup of the IR camera and the typical resonance data due to undamaged and damaged conditions were already investigated in a previous study by the authors [38,49]. An optical laser microphone was placed at a distance less than 50 mm perpendicular to the CF-PEKK specimens (see Figure 2).

### 3.3. Working Principle of the Optical Laser Microphone

**Figure 3:**
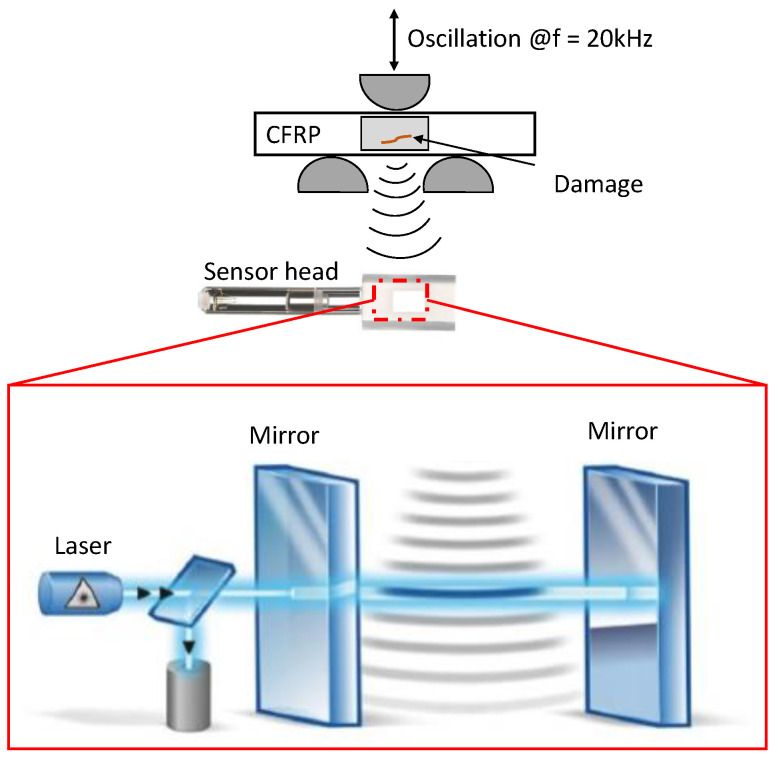
Damage detection technique using optical laser microphone [51].

An optical laser microphone of type ETA100Ultra from Xarion Laser Acoustics GmbH (Vienna, Austria) was used for measuring the acoustic activity due to damage initiation and accumulation during fatigue of CF-PEKK specimens under ultrasonic cyclic three-point bending loading conditions. The membrane-less optical laser microphone operates on the principle of interferometry with a high-frequency bandwidth between 10 Hz and 2 MHz. This measurement system consists of a sensor head, a controller, an amplifier, and a measurement computer [50]. The control unit sends laser light through an optical fiber to the sensor head. The sensor head consists of a pair of parallel, semi-reflective mirrors. During the cyclic loading of the CF-PEKK specimen, the sound pressure around the specimen is influenced by the presence of damage. This change in sound pressure affects the wavelength of light, which is detected optically by changing the refractive index within a Fabry–Pérot etalon. This is shown in Figure 3.

To conduct online measurements, the analog output of the XARION sensor was sampled by a QASS Optimizer4D system from QASS GmbH (Wetter, Germany). It is a measuring system that performs real-time Fast Fourier Transform of the signals using a 24 bit A/D converter and operates up to a sampling rate of 4 MHz.

### 3.4. Ex Situ Damage Characterization Using 3D X-ray Microscopy

Undamaged and damaged CF-PEKK samples at different phases of fatigue life were characterized using X-ray microscopy (XRM) imaging. A Zeiss Xradia Versa 520 system from the Fraunhofer Institute for Applied Solid State Physics (Freiburg, Germany) was used for damage investigation. The XRM consists of

- a sealed transmission X-ray source (30–160 kV),
- a 2 k × 2 k pixel noise-suppressed charge-coupled detector in
- a tunable dual-stage detector system with multiple objectives with different magnifications.

**Figure 4:**
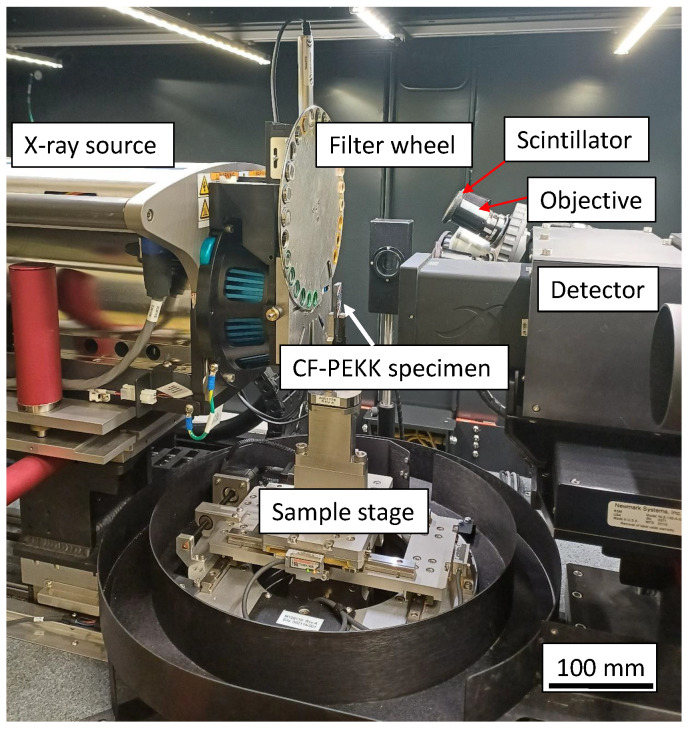
A CF-PEKK specimen mounted inside the Zeiss Xradia Versa 520 3D X-ray microscope (XRM).

In the dual-stage magnification system, the recorded images are enlarged by geometric magnification in the first step. In the second step, a scintillator converts the X-rays into visible light, which is then optically magnified by an optical lens. Combining two stages allows for image capture at a submicron resolution, even for large working distances. A spatial resolution of 1 μm is achievable using the Zeiss Xradia Versa 520 system. The CF-PEKK specimens were mounted as shown in Figure 4.

**Figure 5:**
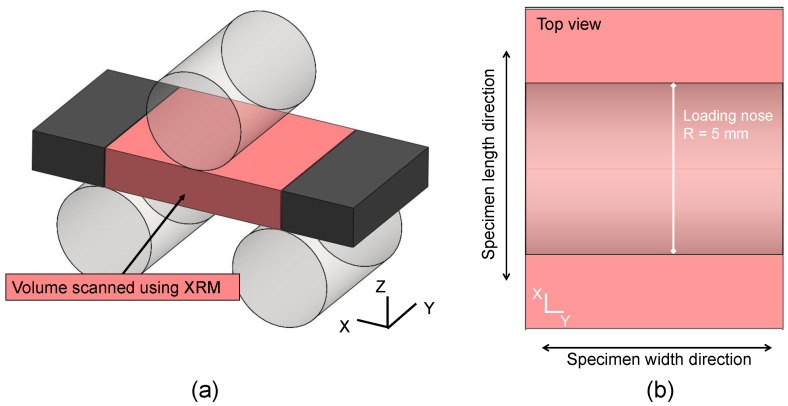
(**a**) A schematic of the CF-PEKK specimens to show the volume scanned using the XRM and (**b**) the top view of the scanned volume of the CF-PEKK specimens representing the length and width directions, as well as the width of loading nose.

A scanning resolution of 10 μm on a volume of 18.6 × 15 × 4.1 mm^3^ (see Figure 5a) took ≈72 h for each specimen scan. All the chosen specimens were scanned once before applying cyclic loading, after experiment interruption, and after failure, thus three times in total. Figure 5b shows the top view of the scanned volume with the area covered by the loading nose to visualize the damaged area from the XRM scan results presented in Section 4.4.

The Zeiss “Scout-and-scan” software (version number: 16.1.14271.44713) was used to control the X-ray measurements. Since the specimens for three-point bending experiments have a flat geometry, the high-aspect-ratio tomography (HART) mode was used. The HART mode allows variable projection distances such that fewer projections are collected along the width and more along the thickness of the specimens.

After performing the X-ray scans, a virtual volume for each specimen was computationally reconstructed from the recorded projection images using the Zeiss XRM reconstruction software (version number: 16.1.14271.44713). A virtual volume is a 3D matrix that segments the sample into voxels. Each matrix element is assigned a grayscale value proportional to the material’s atomic weight in the specimen’s corresponding voxel. For further analysis of X-ray data, ORS Dragonfly Pro software (version number: 2022.2.0.1361) was used [52]. This software was used to visualize the slices of the reconstructed specimen through the reconstructed virtual volume.

### 3.5. Abort Criteria for VHCF Testing at 20 kHz

For displacement-controlled experiments performed using the UFT system, the definition of failure defined in ISO 13003:2003-12 [53] (a standard for determining fatigue properties under cyclic loading) is not applicable as the cyclic loads decrease due to the increase in the compliance (or decrease in stiffness) of the specimens in the presence of damage. Thus, additional failure criteria are required for the UFT systems [54]. The specimen failure or abort (termination) criteria of all the fatigue experiments are given as follows:

- when the maximum surface temperature in any of the defined areas exceeds 80 °C (0.5 × $T_{g}$ of PEKK polymer),
- when the power required by the ultrasonic generator is greater than 700 W as this would mean that there is a significant damping between the resonance unit and specimen, leading to a surface temperature greater than 80 °C, or
- when the resonance frequency drops below 19.5 kHz.

### 3.6. Interrupted Constant Amplitude Fatigue Experiments at 20 kHz

**Figure 6:**
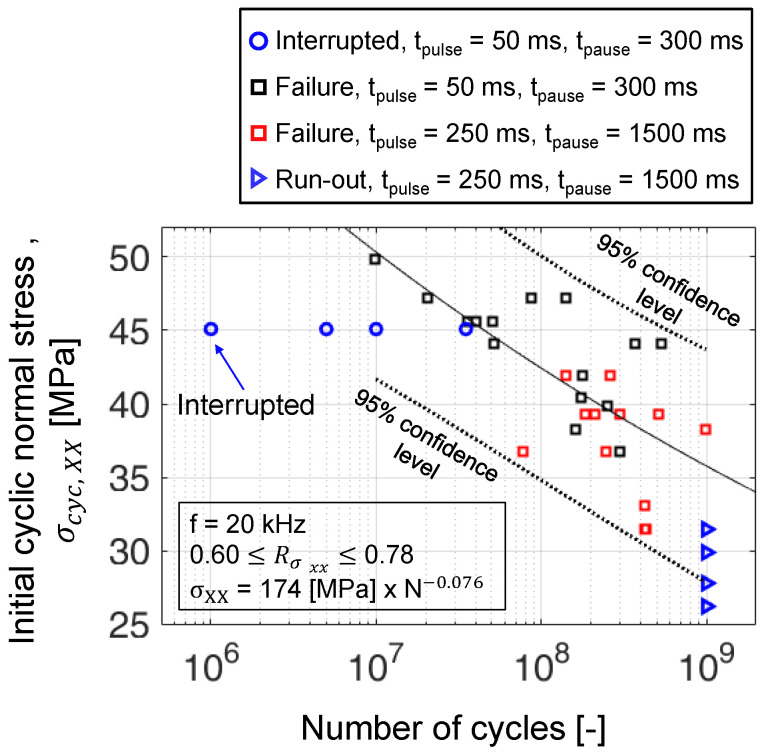
Interrupted CAF experiments denoted with blue circular markers overlaid in the SN diagram previously published in [38].

Four interrupted constant amplitude fatigue (CAF) experiments were performed at a normal stress amplitude of 45.1 MPa. These experiments had pulse and pause durations of 50 ms and 300 ms, respectively. This pulse–pause sequence results in an effective frequency = 2885 Hz. Here, the specimens were interrupted one time, each at different stages of fatigue lives as shown in Figure 6, to measure the change in static stiffness and volumetric damage. At this stress amplitude (45.1 MPa), the maximum time for an experiment surviving 5 × $10^{8}$ cycles (95% confidence limit) is 2 days. Thus, an experimental campaign at a higher stress amplitude was chosen to minimize the overall test time.

The change in static bending stiffness was measured using a table-top static three-point bending test system from ZwickRoell (Ulm, Germany) with a load capacity of 2.5 kN (model name: ZHU/zwickiLine+). The same radius of the loading nose and support pins (radius R = 5 mm) were used with the same span length defined for ultrasonic fatigue experiments. The normalized bending stiffness was obtained by calculating the slope of the force–displacement curves up to a maximum static displacement of 150 μm. The static experiments for stiffness measurements were carried out at a cross-head speed of 1 mm/min as per the ASTM D2344/D2344M-16 standard [55], which is a standard for applying three-point bending loads on short beam composites.

## 4. Results and Discussion

**Figure 7:**
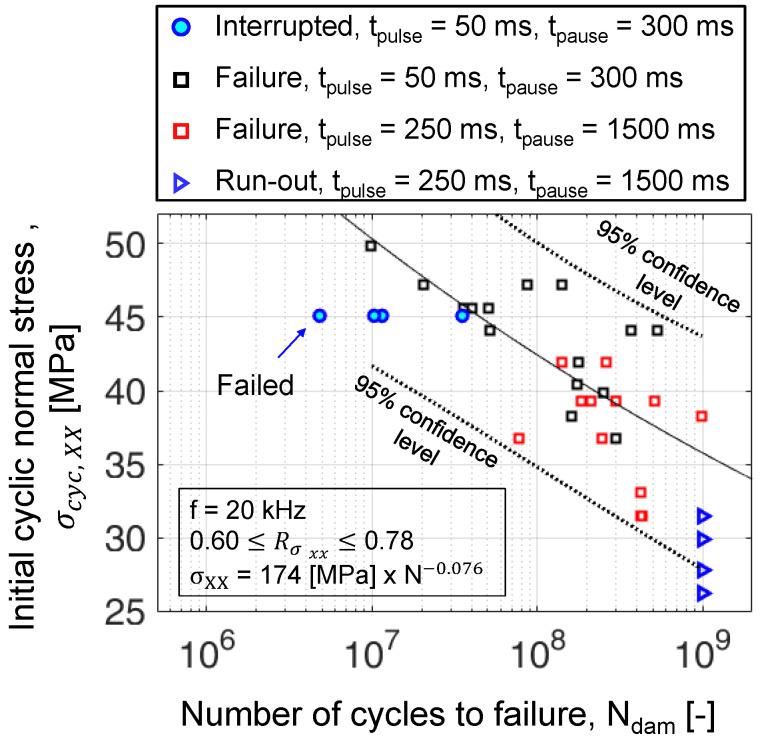
SN diagram with failure of interrupted CAF experiments along with results previously published in [38].

The four CAF experiments at a cyclic stress amplitude of 45.1 MPa and at a frequency of 20 kHz resulted in fatigue lives still within the 95% confidence intervals (see Figure 7). The number of cycles to failure ($N_{dam}$) defined by abort criteria mentioned in Section 3.5 for the four specimens is given in Table 2. Here, $N_{int}$ corresponds to the number of load cycles after which the CAF experiments were interrupted, and $N_{dam}$ refers to the number of load cycles to damage. The fraction between $N_{int}$ and $N_{dam}$ corresponds to the percentage of fatigue life at which the specimens were taken out of the UFT system for residual stiffness and XRM measurements.

### 4.1. Temperature and Resonance Data

**Figure 8:**
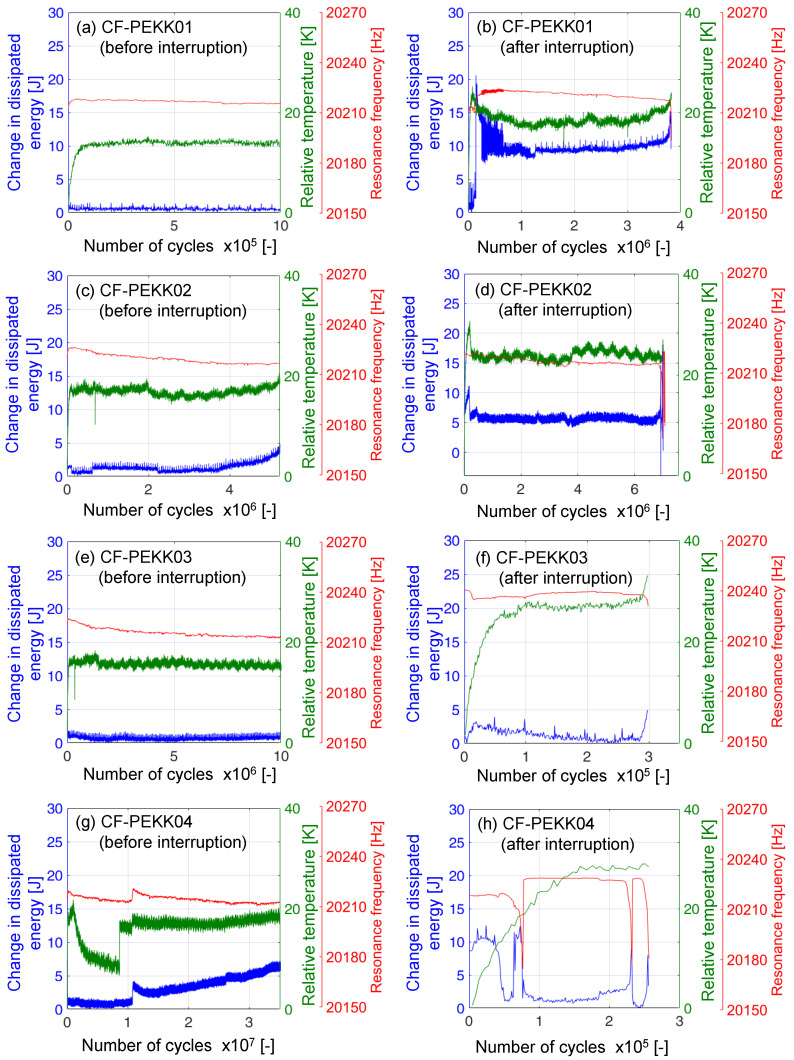
Change in dissipated energy per ultrasonic pulse, relative temperature evolution and drop in resonance frequencies calculated using time–temperature and input resonance signals for the four CF-PEKK specimens tested at $\sigma_{cyc,XX}$ = 45.1 MPa before and after interruption for ex situ damage characterization.

The surface temperature of the CF-PEKK specimens and input resonance signals from the ultrasonic generator were recorded during ultrasonic cyclic loading for all the experiments. The input resonance signals include power expended by the generator, resonance frequency of ultrasonic oscillations, and cyclic displacement amplitude. The temperature and resonance signals were in good agreement during cyclic loading of CF-PEKK specimens in a previous investigation by the authors [49]. From the power expended during every ultrasonic pulse (duration of 50 ms), the energy dissipation can be calculated by determining the area of the power–time curve. Likewise, the peak temperature at the end of each pulse can be calculated from the time–temperature signal. The change in dissipated energy is the difference between the energy dissipated at the end of each pulse and the undamaged condition. The specimens resonate at the desired frequency at undamaged conditions, leading to lesser energy loss. This energy loss increases with increased damping in the presence of damage. In the damaged condition, the energy dissipation and the surface temperature of the specimen increase. The increase in maximum surface temperature at the specimen’s central region since the experiment’s start is denoted as relative temperature. The peak temperature and the energy dissipated at the end of each ultrasonic pulse can be correlated to the fatigue cycles by multiplying the effective frequency (≈1010 Hz) by the number of ultrasonic pulses. These calculations were performed using a Matlab routine, and the results are shown in Figure 8.

The subfigures on the left side (Figure 8a,c,e,g) were recorded before the interruption of the fatigue experiments, and the subfigures on the right side (Figure 8b,d,f,h) were recorded after the interruption of the fatigue experiments until damage. In all these figures, disturbances in the energy loss, resonance frequencies, and relative temperature curves leading to local impulsive peaks could be due to the initiation of any damage, leading to deviation from the ideal resonance conditions. Of all four specimens, CF-PEKK02 and CF-PEKK04 showed an evident increase in energy loss after 4 × $10^{6}$ and 1 × $10^{7}$ cycles, respectively. In contrast, CF-PEKK03 only showed a small gradual drop in resonance frequency before the interruption of the experiment after $10^{7}$ cycles and was found to be damaged after 3 × $10^{5}$ cycles after reload. The relative temperatures on all specimens without damage were less than 20 K for the cyclic normal stress amplitude of 45.1 MPa and increased beyond 20 K due to damage accumulation. Therefore, the end of the ultrasonic fatigue experiment due to failure can be confirmed by a sudden increase in energy loss, surface temperature, or a drop in resonance frequency.

### 4.2. Normalized Drop in Bending Stiffness

**Figure 9:**
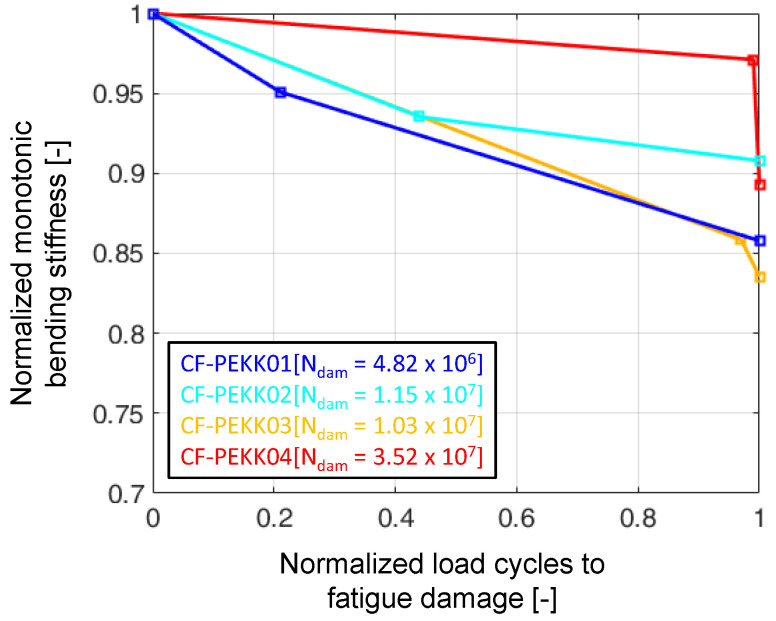
Normalized monotonic (static) bending stiffness drop for the four CF-PEKK specimens due to ultrasonic cyclic three-point bending at $\sigma_{cyc,XX}$ = 45.1 MPa.

In the literature, the stiffness drop due to fatigue damage in composites is attributed to the initiation, accumulation, and propagation of multiple transverse cracks [2,11,30,56,57,58]. The normalized drop in monotonic (static) bending stiffness is plotted against normalized cycles to fatigue failure in Figure 9. Here, the CF-PEKK01 specimen, taken out of the UFT system after 21% of fatigue life, shows the maximum drop at the 20% mark (about 5% stiffness drop), leading to the shortest fatigue life to failure. Similarly, the CF-PEKK04 specimen taken out after 99% of fatigue life shows the least drop in stiffness (about 3%), leading to the longest fatigue life. CF-PEKK02 and CF-PEKK03 specimens had an equal stiffness drop of about 7% after 50% of fatigue life, leading to closer fatigue lives. Since the abort criteria also include a temperature limit of 80 °C, which can also be localized heating in the vicinity of damage, the interruption of the fatigue experiment depends on the side of the specimen where damage accumulated. This means damage can accumulate near the surface monitored by the IR camera or near the opposite surface, leading to a slightly different severity in damage accumulation until the experiment is aborted. This difference in the accumulation of damage leads to different stiffness drops ranging from 10 to 20%. An experiment leading to up to 20% stiffness drop occurs when the damage occurs on the opposite side of the specimen, which is not monitored by the IR camera.

Looking back at Figure 8a, the relative temperature, energy loss, and resonance frequency signals do not have any disturbance up to $10^{5}$ cycles. However, the stiffness drop is the maximum of all experiments at their 20% fatigue life. One reason for this could be the development of multiple microscopic damage mechanisms that only reduce the global stiffness of the specimen but are not captured by the temperature and input resonance signals. In other words, the infrared thermography and resonance data are inadequate to capture the microscopic damage observed in CF-PEKK01 before interruption. After reloading CF-PEKK01 with the same loading parameters, the temperature and input resonance signals detect further development of microscopic damage (see Figure 8b). This can be seen from a strong signal disturbance within the first $10^{6}$ cycles. A similar trend can also be seen in the case of the CF-PEKK03 specimen, where the resonance frequency drops by ≈10 Hz, corresponding to a stiffness drop of 15% until test interruption (at 97% fatigue life). After reloading the specimen, the relative temperature increased from 15 K to 27 K (see Figure 8e,f). Thus, the relative temperature and input resonance signals present an accurate damage condition in the composite specimens when examined together with the residual stiffness.

If these four specimens can be collectively considered as representative of the residual stiffness change during ultrasonic fatigue testing, one could say that the stiffness drops steeply within 20% of fatigue life, after which a gradual degradation is seen up to 97%. Between 97 and 100% of fatigue life, the residual stiffness drops strongly, leaving the specimen’s state to satisfy one of the abort criteria described for UFT.

### 4.3. Observations from Results Obtained Using the Optical Laser Microphone

**Figure 10:**
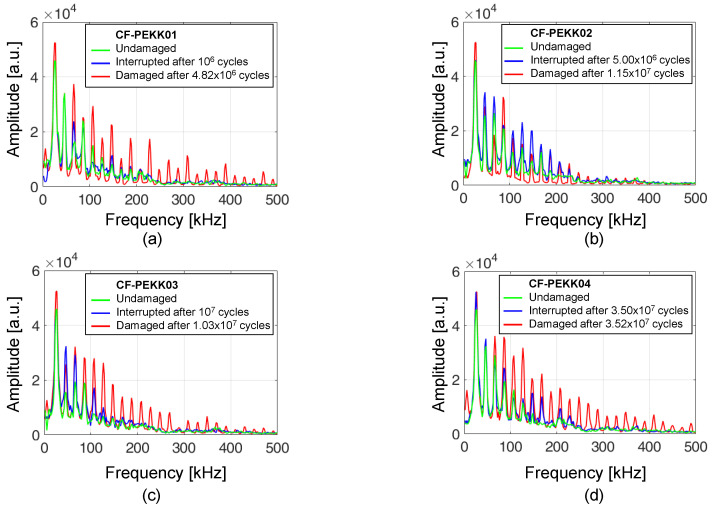
FFT spectra of 4 CAF experiments interrupted at (**a**) $10^{6}$ cycles, (**b**) 5 × $10^{6}$ cycles, (**c**) $10^{7}$ cycles, and (**d**) 3.5 × $10^{7}$ cycles.

Figure 10a–d shows the Fast Fourier Transform (FFT) spectra of the four CF-PEKK specimens obtained using the optical laser microphone at different stages of fatigue for a normal stress amplitude of 45.1 MPa. The first tallest peak in Figure 10a–d corresponds to the loading or the excitation frequency of the UFT system. The tiny peak before 20 kHz is recorded due to the sound of the compressed air cooling setup. Several smaller amplitude (higher harmonics) peaks follow the highest peak at 20 kHz. About 7–9 peaks up to frequencies less than 200 kHz can be seen for undamaged CF-PEKK specimens (green curves). The peak count is about 22–24 and can even be observed up to 500 kHz for damaged specimen conditions (red curves). This increase in the number of peaks from undamaged to damaged conditions could be caused due to friction between the damaged surfaces. In other words, the higher harmonics could indicate the presence of the so-called breathing cracks in the oscillating structure [59]. These cracks open and close during cyclic oscillations [60]. Such a scenario in woven composites could occur with transverse cracks before growing into delamination [10,57,61] along the weft fiber bundle direction.

For the CF-PEKK specimen interrupted after 21% of its fatigue life (Figure 10a), the peaks in the interrupted FFT signal almost overlap with the FFT signals of the undamaged state. This marks the absence of macroscopic damage accumulation at about 20% fatigue life. The peaks in the FFT signal of the CF-PEKK specimen interrupted after 44% of fatigue life (Figure 10b) overlap mostly with the peaks in the FFT signals of the damaged state. In contrast, for the CF-PEKK specimens interrupted after 97 and 99% of fatigue lives, the peaks of the FFT signals from the damaged state are more pronounced than the interrupted states. The peaks in the interrupted state for CF-PEKK03 and CF-PEKK04 do not align completely with the undamaged state, suggesting the potential presence of damage, which can be described using the XRM observations.

### 4.4. X-ray Microscopy (XRM) Observations

**Figure 11:**
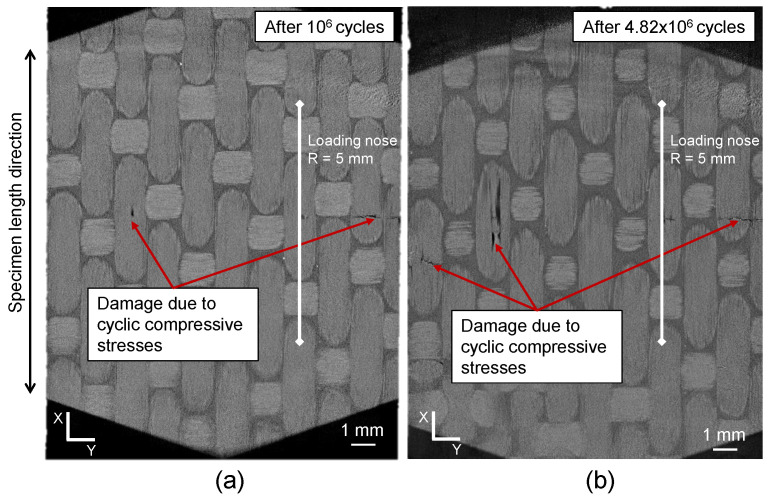
XRM images of CF-PEKK01 specimen showing the state of damage from the top view after (**a**) 21% and (**b**) end of fatigue life.

The XRM analysis of CF-PEKK01 showed that damage initiated at the top side of the specimen due to local compressive stresses after 21% fatigue life, which failed after 4.82 × $10^{8}$ cycles at the same location. Figure 11a shows the only locations where damage was found across the specimen volume. This damage was found on the top layer of the CF-PEKK specimen, which is also represented as a schematic in Figure 5b. This damage location is typical for short beam shear specimens [38,45]. Since this damage initiation location is away from the specimen surface monitored using the IR camera, this damage is not detected by the temperature signals in Figure 8a. The final damage after specimen failure was an accumulation of longitudinal and transverse cracks across the top layer of the CF-PEKK01 specimen (see Figure 11b). A transverse crack can be seen to propagate to the specimen edge monitored using thermography and thus captured in the relative temperature signal in Figure 8b.

**Figure 12:**
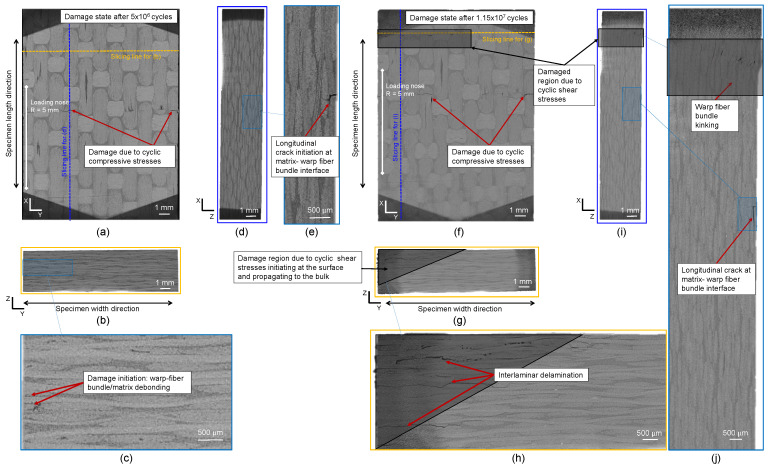
XRM images of the CF-PEKK02 specimen showing the state of damage from the (**a**) top view, (**b**) a slice across the YZ plane, (**c**) the zoomed-in view showing damage initiation in the YZ plane, (**d**) a slice across the XZ plane, (**e**) the zoomed-in view showing damage initiation in the XZ plane after 44% fatigue life, (**f**) the top view, (**g**) a slice across the YZ plane, (**h**) the zoomed-in view showing damage propagation in the YZ plane, (**i**) a slice across the XZ plane and (**j**) the zoomed-in view showing damage propagation in the XZ plane of the specimen after failure.

Figure 12 shows the results of XRM scans after interruption and damage for the CF-PEKK02 specimen. Figure 12a corresponds to the top view of the CF-PEKK specimen after 5 × $10^{6}$ cycles. Figure 12b is obtained by slicing the specimen along its width, and Figure 12c is a zoomed-in view of this sliced plane showing damage initiation near the edge of the CF-PEKK specimen. Figure 12d is obtained by slicing the specimen along its length as shown by slicing lines in Figure 12a,e depicting the zoomed-in view showing longitudinal crack initiation at the matrix-warp fiber bundle interface. At the interrupted state, damage due to compressive stresses directly under the loading nose can be seen in Figure 12a,d,e. A slice along the width of the specimen shows damage initiation at the edge of the specimen due to cyclic shear stresses with fiber bundle/matrix debonding (Figure 12b,d). The XRM scan performed after the end of the fatigue experiment (after 1.15 × $10^{7}$ load cycles) on the same specimen (CF-PEKK02) showed severe damage in the same locations spotted during the interrupted scan. This severity is marked with gray shaded regions in Figure 12f–j. Due to cyclic shear stresses, warp fiber bundle kinking was observed in the region between the support unit and loading nose (Figure 12f,i,j). The damage initiation observed near the edge of the CF-PEKK specimen in Figure 12b,c was found to propagate into the bulk of the material (see Figure 12g,h). The damage propagated to about two-third of the width of the specimen from the edge to the bulk. This can be seen in Figure 12h with damage features such as transverse interlaminar delamination.

**Figure 13:**
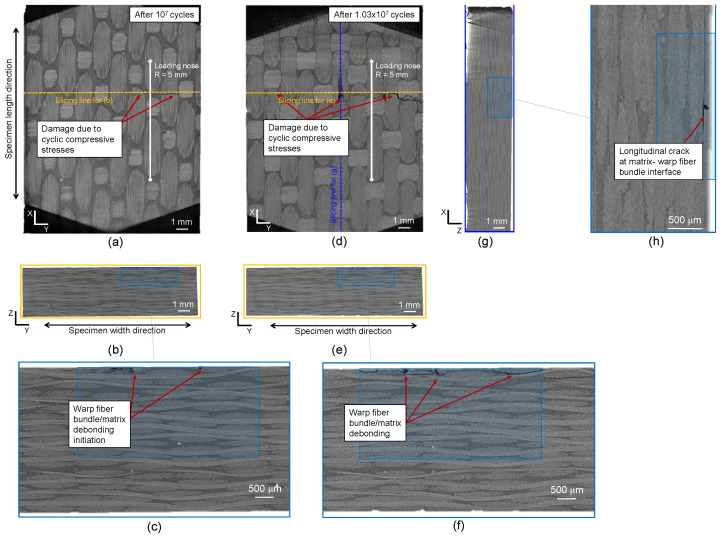
XRM images of the CF-PEKK03 specimen showing the state of damage from the (**a**) top view, (**b**) a slice across YZ plane, (**c**) the zoomed-in view to show damage initiation in the YZ plane after 97% fatigue life, (**d**) the top view, (**e**) a slice across the YZ plane, (**f**) the zoomed-in view to show damage propagation in the YZ plane, (**g**) a slice across the XZ plane and (**h**) the zoomed-in view to show damage propagation in the XZ plane of the specimen after failure.

For the CF-PEKK03 specimen, the damage was observed in the top layer (see Figure 13a). Here, the warp fiber bundles were found to debond from the PEKK matrix (see Figure 13b,c), leading to transverse cracks after $10^{7}$ that grew longer, leading to failure after 1.03 × $10^{7}$ cycles (see Figure 13d,e). Here, the damage initiated at the top layer and propagated within the same layer as warp fiber bundle debonding. Such a damage mechanism could have resulted in a small resonance frequency drop in Figure 8e. However, since this damage occurred away from the specimen surface monitored by the infrared camera, it was not captured by the temperature signals. Only the final failure leading to a transverse crack up to the edge of the specimen was captured by the IR camera in Figure 8f. A longitudinal crack was found (see Figure 13g,h) when the specimen was sliced along the XZ plane near the specimen center. Until this crack development, no change in energy loss could be observed in Figure 8f, meaning the failure was sudden.

**Figure 14:**
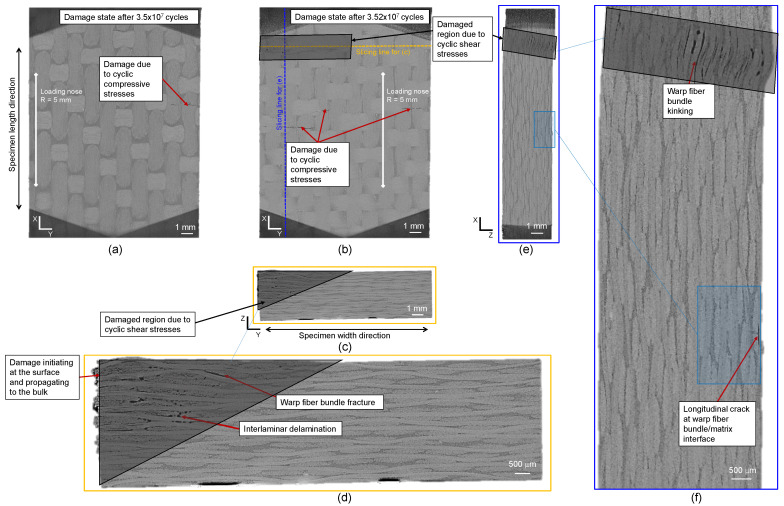
XRM images of the CF-PEKK04 specimen showing the state of damage from the (**a**) top view after 99% fatigue life, (**b**) the top view, (**c**) a slice across the YZ plane, (**d**) the zoomed-in view showing damage propagation in the YZ plane, (**e**) a slice across the XZ plane and (**f**) the zoomed-in view showing damage propagation in the XZ plane of the specimen after failure.

The XRM scans of the CF-PEKK04 specimen showed the absence of any macroscopic damage due to shear stresses up to 99 % of fatigue life, with only a single crack observed on the top of the specimen due to local compressive stresses from the loading nose (see Figure 14a). The XRM scans after the end of the fatigue experiment (Figure 14b–f) showed damage morphologies similar to the damaged state of CF-PEKK02, meaning the damage severely accumulated in the last percent of the fatigue life. These results align with those obtained from the FFT spectra for interrupted and damaged conditions of the CF-PEKK04 specimen. Here, warp fiber bundle kinking was observed due to cyclic shear stresses and longitudinal cracks below the top layer due to local compressive stresses.

### 4.5. Discussion on the Results Obtained Using Optical Laser Microphone and XRM

The damage initiates at the surface due to the free edge effect [62,63] and accumulates across the width of the specimen in the form of transverse cracks [64]. In five-harness satin fabric architecture, transverse cracks are off-axis cracks that initiate in the matrix and propagate along the weft bundle direction, leading to weft fiber bundle delamination (see Figure 12c,h) [30]. Now, the definition of crack initiation in composite materials is even harder to define than in metals and other homogenous materials. Reifsneider (1980) described fatigue in composites as a sequential damage development with crack nucleation in off-axis plies. This could be a transverse crack. However, damage initiation can be identified at micro- and macroscopic levels [65]. Caiulo and Kachanov (2010) [66] reported that there is no correlation between the clustering of microcracks and stiffness reduction at the structural level. This is why the definition of Salkind (1972) [67] for damage initiation as the crack of detectable size seems more practical but dependent on the damage monitoring system.

The higher-order harmonics due to damage during ultrasonic fatigue testing were already investigated using signals from a single-point Laser Doppler Vibrometer and optical laser microphone [68]. In another study, acoustic signals from a contactless optical laser microphone were used for non-linear analysis of damage evolution in CFRP during VHCF [69]. Studies on material non-linearity parameters are not new. A substantial monotonic increase in material non-linearity of Ti-6-Al-4V due to fatigue loading was reported in [70,71]. Later, this approach was used to detect early damage detection in CFRP [69,72]. However, the damage cannot be quantified when the optical laser microphone is the only in situ damage characterization technique. These results are much more useful when correlated with another technique that quantifies the shape and size of damage. Furthermore, each higher harmonic peak shown in Figure 10 should be matched to a specific damage feature to predict the damage state inside the composite specimen by just observing the FFT spectra during ultrasonic loading. Ex situ XRM scans are useful to compare with the results obtained using the optical laser microphone. Only macroscopic damage could be captured in this investigation since the CF-PEKK specimens are scanned using XRM without static load. This challenge could be overcome by performing in situ XRM measurements or applying static loading through a small fixture during XRM scans. Neither of these options was immediately possible with the current 3D XRM system at the Fraunhofer IAF facility. Furthermore, a higher resolution (in the order of 1 μm) scan could provide more information on the microscopic damage features. However, since damage can occur anywhere in the volume between the support pins (18.6 × 15 × 4.1 mm^3^), the scan time would also increase significantly (×100). A higher resolution scan in selected areas where damage can occur is proposed for future investigations. Nevertheless, combining these two measurement techniques provides two important observations.

- The damage accumulation rate can be different even if the loading parameters are the same depending on the interaction between the local stress state at the weakest links in the composite material.
- Damage due to cyclic compressive stresses and the stress concentration at the contact between the loading nose and specimen occurs at the top layer of the CF-PEKK specimens. A longitudinal crack results from the accumulation of weft fiber bundle-matrix debonding.
- The damage due to cyclic shear stresses initiates at the surface due to the free-edge effect and progresses to the bulk of the specimen.

**Figure 15:**
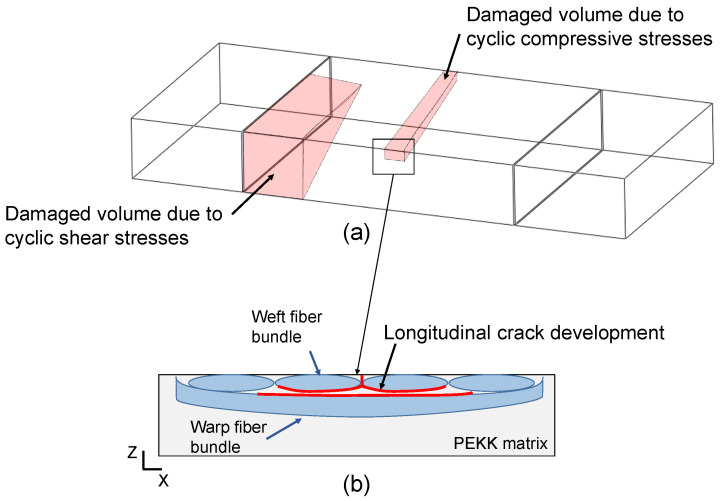
(**a**) A schematic of the CF-PEKK specimen showing damage sites observed using XRM and (**b**) a sketch of the longitudinal crack.

The different damage accumulation rates result in different fatigue lives for specimens tested at the same loading conditions. Due to cyclic compressive stresses and stress concentration between the loading nose and specimens, the damage accumulation occurs in the form of longitudinal cracks in the XZ plane (see Figure 15b) and transverse cracks in the YZ plane, both debonding the warp fibers from the PEKK matrix. The damage due to cyclic shear stresses was found to occur in one of the four regions, between the support pins and loading nose, where high positive and negative shear stresses are witnessed. Figure 15a shows a representative schematic of the damaged volume due to cyclic shear stresses. The damage initiates at the surface and progresses towards the bulk of the material.

The final damaged state results from accelerated structural degradation due to damage accumulation resulting in localized surface temperature reaching 80 °C or when the specimen loses its ability to resonate. However, for CF-PEKK specimens, damage occurred at different locations on the surface due to high tensile, compressive, or shear stresses and their interaction with the weakest links [38] since they are short beams loaded with shear and bending forces. This means the damage accumulation rate depends on the location of damage initiation and the local stresses around it.

The final failure morphologies in Figure 12h and Figure 14d show the saturation of cracks near the surface and propagation into interlaminar (or inter-ply) delamination. This observation is essential for ultrasonic fatigue testing of composites. If the temperature in the bulk of the specimen is much higher than the temperature at the surface, the damage will also be adverse due to the softening of the polymer material. In other words, if a surface temperature of 80 °C were measured using an IR camera, a higher temperature in the bulk of the material would mean increased damage accumulation in the bulk. In contrast, Figure 12h and Figure 14d show that the damage due to shear stresses initiates at the edge and accumulates only up to about two-third of the width of the specimens, and the damage population decreases from the edge to the bulk. This means that the temperatures at the bulk of the CF-PEKK specimens cannot be much higher than at the edges of the specimens. Therefore, assessing damage on the surface of the composite specimens loaded under ultrasonic cyclic three-point bending using thermography and digital light optical microscopy [49] would provide valid results.

## 5. Summary and Conclusions

Thermography provides essential insights into damage accumulation that occurs at the surface of composite specimens and is extensively used in the fatigue characterization of composites. An important question to be addressed for ultrasonic fatigue testing of composites is the state of damage in the bulk of the material and the possibility of temperature influence due to friction between the damaged surfaces. Furthermore, only one side of the specimen is usually monitored using the infrared (IR) camera. This prompts another question: how can thermography be used to reliably capture the fatigued condition of the composite specimens if damage occurs either in the bulk or on the opposite side of the specimen, which is not covered by thermography?

Macroscopic damage anywhere in the composite changes the resonance characteristics (energy loss by ultrasonic generator and resonance frequency), which was reflected in the thermal response of the specimen with increased heating. This increased heating of the specimens occurs due to the loss of the CF-PEKK specimen’s capacity to oscillate at ideal resonance conditions due to damage accumulation. Therefore, the surface characterization (thermography and light optical microscopy) could be related to the global properties (dynamics response and residual stiffness) of the CF-PEKK specimens. However, the damage state in the bulk of the material is still unknown.

This investigation mapped the damage initiation and accumulation locations using 3D X-ray microscopy (XRM). By looking into the volume of the damaged specimen, it was possible to relate the physical damage to the observed thermal and dynamic response of the CF-PEKK material. Furthermore, XRM provides strong evidence that the thermal influence on fatigue damage in composites due to ultrasonic oscillations can be minimized or even avoided with suitable ultrasonic test parameters (pulse duration, pulse–pause ratio) that lead to a surface temperature below 0.5 × $T_{g}$ [°C] of the polymer matrix. This can be confirmed with damage population near the surface rather than in the bulk of the CF-PEKK specimens.

Finally, with the use of an optical laser microphone, it was observed that damage in the composite specimen affects not only the eigenmode frequency but also the higher harmonics of the eigenmode frequency. However, more explanation for the change in amplitude and peak count of these higher harmonics needs to be further investigated to use such a technology for online damage assessment during ultrasonic fatigue testing of fiber-reinforced composites.

From this investigation, the following conclusions were drawn:

- The very high cycle fatigue damage in a CFRP specimen under ultrasonic cyclic three-point bending, due to shear stresses, initiates and populates at the surface.
- The temperature in the bulk of the specimen cannot be much higher than the temperature measured at the surface using infrared thermography.
- The scatter in the fatigue life of composites under cyclic three-point bending is due to the different damage initiation sites and the local stress state in the vicinity of initiated damage, leading to different damage accumulation rates.
- A combination of different measurement techniques is required to adequately capture the damage accumulation in fiber-reinforced polymers under ultrasonic cyclic three-point bending.

## Figures and Tables

**Table 1:** Properties of CF-PEKK composite laminate previously published in [45].

Elastic Properties	Values
Fiber architecture	5H satin weave fabric
Fiber volume fraction [%]	50
Consolidated ply thickness [mm]	0.31 ± 0.01
Number of plies [-]	13
Composite layup [-]	[0/90/0/90/0/90/0]$s_{\;}$
Glass transition temperature [°C]	* 160
Melting temperature [°C]	* 337
Tensile modulus 0° [GPa]	58.0
Ultimate tensile strength 0° [MPa]	** 776
Ultimate compressive strength 0° [MPa]	** 585

Values obtained from material data sheets provided by the manufacturer, [43] * and [44] **.

**Table 2:** Load cycles to interruption and damage for CAF experiments conducted at $\sigma_{cyc,XX}$ = 45.1 MPa.

Specimen ID	$N_{\mathrm{int}}$	$N_{\mathrm{dam}}$	$N_{\mathrm{int}}/N_{\mathrm{dam}}$
	[-]	[-]	%
CF-PEKK01	$10^{6}$	4.82 × $10^{6}$	21
CF-PEKK02	5 × $10^{6}$	1.15 × $10^{7}$	44
CF-PEKK03	$10^{7}$	1.03 × $10^{7}$	97
CF-PEKK04	3.5 × $10^{7}$	3.52 × $10^{7}$	99

## Data Availability

Data are contained within the article.
